# Computational and Experimental Insights into the Mechanism of Substrate Recognition and Feedback Inhibition of Protoporphyrinogen Oxidase

**DOI:** 10.1371/journal.pone.0069198

**Published:** 2013-07-23

**Authors:** Ge-Fei Hao, Ying Tan, Sheng-Gang Yang, Zhi-Fang Wang, Chang-Guo Zhan, Zhen Xi, Guang-Fu Yang

**Affiliations:** 1 Key Laboratory of Pesticide & Chemical Biology of Ministry of Education, College of Chemistry, Central China Normal University, Wuhan, P. R. China; 2 State Key Laboratory of Elemento-Organic Chemistry, Nankai University, Tianjin, P. R. China; 3 Department of Pharmaceutical Sciences, College of Pharmacy, University of Kentucky, Lexington, Kentucky, United States of America; University of Akron, United States of America

## Abstract

Protoporphyrinogen IX oxidase (PPO; EC 1.3.3.4) is an essential enzyme catalyzing the last common step in the pathway leading to heme and chlorophyll biosynthesis. Great interest in PPO inhibitors arises from both its significance to agriculture and medicine. However, the discovery of PPO inhibitors with ultrahigh potency and selectivity is hampered due to lack of structural and mechanistic understanding about the substrate recognition, which remains a longstanding question central in porphyrin biology. To understand the mechanism, a novel binding model of protogen (protoporphyrinogen IX, the substrate) was developed through extensive computational simulations. Subsequently, amino acid residues that are critical for protogen binding identified by computational simulations were substituted by mutagenesis. Kinetic analyses of these mutants indicated that these residues were critical for protogen binding. In addition, the calculated free energies of protogen binding with these mutants correlated well with the experimental data, indicating the reasonability of the binding model. On the basis of this novel model, the fundamental mechanism of substrate recognition was investigated by performing potential of mean force (PMF) calculations, which provided an atomic level description of conformational changes and pathway intermediates. The free energy profile revealed a feedback inhibition mechanism of proto (protoporphyrin IX, the product), which was also in agreement with experimental evidence. The novel mechanistic insights obtained from this study present a new starting point for future rational design of more efficient PPO inhibitors based on the product-bound PPO structure.

## Introduction

To understand the function of an enzyme, the binding of the substrate (S) and the structure of the enzyme–substrate (ES) complex are central issues in determining the catalytic mechanism. The structural information obtained from the ES complex at the atomic level is required for understanding the substrate recognition mechanism of the enzyme. However, due to the enzyme’s catalytic transformation of substrate into product, it is often difficult to grow an intact crystal and to determine the crystal structure of the ES complex. New approaches to examine the binding model of the substrate and the enzymatic mechanism are topics of intense investigation in current enzymology.

**Figure 1:**
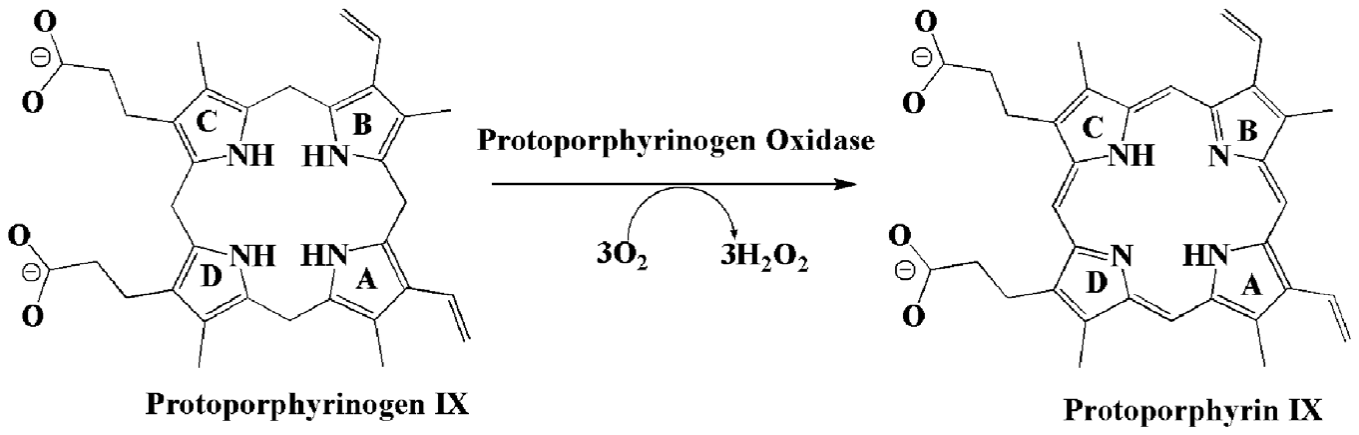
Protoporphyrinogen oxidase (PPO) catalyzes the oxidation reaction of protoporphyrinogen IX (protogen) (left) with molecular oxygen to produce protoporphyrin IX (proto) (right). In this reaction, the molecular oxygen is reduced to hydrogen peroxide, and six hydrogen atoms are eliminated.

Tetrapyrroles, such as chlorophyll, heme, bilins, and porphyrins, play pivotal roles in electron transfer-dependent energy generating processes including photosynthesis and respiration of all biological systems [1]. One of the steps of tetrapyrrole biosynthesis rely on protoporphyrinogen IX oxidase (PPO; EC 1.3.3.4), which catalyzes the six-electron oxidation of protogen (protoporphyrinogen IX, the substrate) to proto (protoporphyrin IX, the product) in both eukaryotic and prokaryotic organisms (**Figure 1**) [2], [3]. PPO plays a significant and pivotal role in the life cycle by the oxidation of protogen which is a prerequisite step for the synthesis of heme and chlorophylls and thus the molecular mechanism associated with the substrate recognition by PPO has attracted great interest in recent decades [4], [5], [6], [7], [8].

The inhibition or functional loss of PPO results in the accumulation of protogen, which can be spontaneously oxidized to proto by oxygen. As a photosensitizer, in the presence of light, proto can further induce the production of singlet oxygen, causing lipid peroxidation and cell death [9]. Thus, PPO is considered an important target for herbicide discovery [10], [11] and may be a focus for bactericide and fungicide design [5], [12]. Inhibitors of PPO may also find application in cancer treatment through photodynamic therapy (PDT) [13], [14], [15]. The discovery of PPO inhibitors with high potency and selectivity has been hampered by the lack of structural and mechanistic understanding of substrate recognition. Due to its spontaneous oxidation and the instability of protogen, determination of the crystal structure of the substrate-bound PPO has not been achieved.

Due to the extremely high time resolution and atomic level representation, computational simulation has been increasingly used in understanding the detailed mechanism of substrate recognition in proteins [16], [17]. Hence, in the present study, extensive computational simulations and experimental techniques were integrated to discover the binding model and detailed recognition mechanism of the substrate by PPO. We used conformational analysis based molecular docking, molecular dynamics (MD) simulation, molecular mechanic/Poisson-Boltzmann surface area (MM/PBSA) calculations, umbrella sampling MD simulations, and potential of mean force (PMF) calculations. We also introduced mutations into PPO by site-directed mutagenesis and then conducted enzyme kinetics studies. A proposed novel mechanism for substrate recognition and product feedback inhibition by PPO based on computational simulation was in agreement with site-directed mutagenesis and enzyme kinetic study. This is the first report of the substrate recognition and feedback inhibition mechanism of PPO that combines theoretical and experimental methods. The demonstrated structural and energetic profiles provide a new starting point for future structure- and product-based design of highly potent PPO inhibitors for the development of agrochemicals or PDT cancer therapy.

## Results and Discussion

### Identification of the Substrate Binding Model

Molecular docking studies were performed to simulate the binding of protogen to PPO. However, conventional docking methods do not take into account the flexibility of macrocyclic compounds. So, an extensive conformational analysis of protogen was performed to obtain a total of 18 conformers (M1–M18, see details in **Figure S1**), all of which were used as starting structures for docking.

According to the binding affinities determined by Autodock4, conformers M2 (−11.03 kcal/mol), M14 (−10.99 kcal/mol), M15 (−11.88 kcal/mol), M16 (−12.48 kcal/mol), M17 (−11.20 kcal/mol), and M18 (−11.18 kcal/mol) should be the most feasible structures. To avoid the limitations of the Autodock score, MM/PBSA calculations were performed, identifying conformers M6 (−26.32 kcal/mol), M7 (−35.62 kcal/mol), M9 (−29.98 kcal/mol), M10 (−42.73 kcal/mol), M14 (−44.47 kcal/mol), and M15 (−35.66 kcal/mol) as the more favorable ligand conformations. The ligand must always undergo deformation from the solution conformation to the binding conformation. Additionally, the conformational energy penalty for the bioactive conformations should be less than 6 kcal/mol [18]. We therefore calculated the conformational energy penalty of each conformer by using Gaussian 03 at 6-31+G* basis set with the B3LYP method plus PCM solvation model. In addition, at the active site, the methylene bridge atom in the *meso* position of protogen and the N_5_ atom of the FAD (**Table S2**) should be close enough to each other to produce the correct reaction orientation. The potential binding models were ultimately identified through the overall evaluation of the docking score of Autodock, MM/PBSA calculations, reaction orientations, and conformational energy penalties (See details in **Table S1** and **Table S2**). Additionally, to avoid the shortcomings of the Autodock program and the conformational analysis method, we tested other docking programs (*i.e.,* Gold) with different starting structures and obtained very similar results (see details in **Figure S2**). Ultimately, conformers M14 and M15 were selected as potential binding models for further analysis. Particularly, M14 was similar with the binding model proposed by Koch et al. [19].

Since the docking algorithm did not fully account for the structural flexibility of the protein, we performed MD simulations for M14 and M15, using PPO from *Nicotiana tabacum* mitochondria (*mt*PPO). According to the analysis of RMSD and some key distances of the MD trajectories (**Figure S3**), the simulations reached equilibrium states. Further, the MD simulations of M14 and M15 were also repeated with different sets of parameters and force-fields to validate the convergence of the simulations (see details in **Figure S4**). The binding model associated with stronger hydrogen bonding networks was the most reasonable. Hence, the strengths of the hydrogen bonding networks of M14 and M15 were evaluated by performing HBE (hydrogen bond energy) calculation and hydrogen bond distance analysis (summarized in **Table 1** **)**. For M14, the carbonyl oxygen atoms of the propionate group formed up to two hydrogen bonds with R98 (N–H…O) and one hydrogen bond with S235 (O–H…O) in tobacco *mt*PPO. The total HBE is −12.0 kcal/mol. For M15, however, the carbonyl oxygen atoms of the protogen propionate group formed up to three hydrogen bonds with R98 (N–H…O) and the pyrrole cycle formed two hydrogen bonds with G175 (N–H…O) in tobacco *mt*PPO. The total HBE is −17.4 kcal/mol. Further, the binding free energy of M15 was much lower than that of M14 based on a large amount of MD sampling (**Table S2**). Although the conformational energy penalty of M15 was slightly higher than M14, the reaction orientation of M15 was more advantageous than that of M14 (**Table S2**). These computational results suggest that M15 should be a significantly more favorable binding conformation than M14 (**Table 1**).

**Table 1:** Hydrogen bond networks of the M14 and M15 conformations in complex with tobacco *mt*PPO.

Bind Model	Acceptor	Donor	Mark	%^a^	Max^b^	Min	Ave	HBE^c^	THBE^d^
M14	TEM^e^	S235	D1	99.8	3.1	1.5	1.8 (0.16)	−1.9 (0.24)	−12.0
	TEM	R98	D2	98.3	3.1	1.6	1.9 (0.19)	−6.2 (0.50)	
	TEM	R98	D3	92.5	3.4	1.7	2.2 (0.22)	−3.9 (0.46)	
M15	G175	TEM	D1	93.3	2.9	1.7	2.4 (0.16)	−2.0 (0.24)	−17.4
	G175	TEM	D2	82.8	3.4	1.9	2.5 (0.23)	−1.6 (0.23)	
	TEM	R98	D3	99.7	2.8	1.6	1.9 (0.13)	−6.8 (0.40)	
	TEM	R98	D3	91.1	3.2	1.7	2.3 (0.29)	−2.9 (0.46)	
	TEM	R98	D3	84.7	3.7	1.6	2.2 (0.39)	−4.0 (0.58)	

[a] Occupancy of hydrogen bonds (The occupancy >70% were listed).

[b] Hydrogen bond distance (Å).

[c] Hydrogen bond energy (kcal/mol), calculated according to equation: 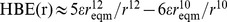, the parameters: r_eqm_ = 1.8 Å, ε = 8.4 kcal/mol. We calculated the HBE of every snapshot of the MD simulation and then took the average value, as in our previous studies [36], [46], [47], [48]. The values in the parentheses are the standard deviations.

[d] Total hydrogen bond energy (kcal/mol). The total HBE value is the average of the HBE values calculated by using the instantaneous distances in all of the snapshots.

[e] The substrate name in pdb file.

**Figure 2:**
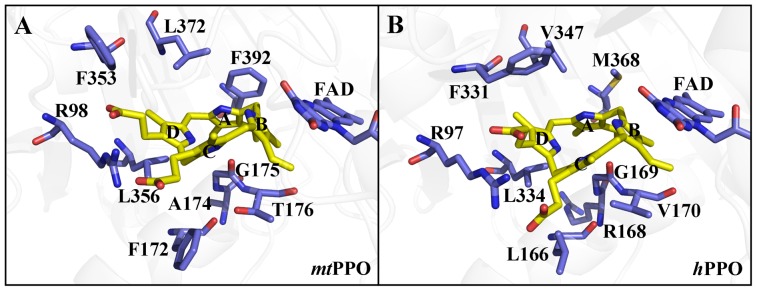
The binding models of protogen in tobacco *mt*PPO and *h*PPO. (**A**) Side view of protogen surrounded by R98, F172, A174, G175, T176, F353, L356, F392, and FAD in tobacco *mt*PPO. (**B**) Side view of protogen surrounded by R97, L166, R168, G169, V170, F331, L334, M368, and FAD in *h*PPO.

Based on the identified binding model (M15) of protogen in tobacco *mt*PPO, the surrounding amino acid residues that interacted with rings A, B, C, and D of protogen were further analyzed. Ring A of protogen interacted with A174 and was kept in position by π-π stacking interaction with F392 (**Figure 2A**). Ring B of protogen was inserted into a slot of T176 and made π-π stacking interaction with cofactor FAD. Ring C of protogen interacted with F172 and G175, while ring D interacted with residues F353 and L356. In addition, the propionate groups of both rings C and D could form hydrogen bonds with R98 in tobacco *mt*PPO.

The computationally identified novel binding model was consistent with available experimental data obtained from site-directed mutagenesis studies on tobacco *mt*PPO [20]. According to the simulated binding model, conserved residues F392, L356, L372, and R98 are essential for protogen binding. The apparent *K*_m_ value of purified wild-type tobacco *mt*PPO for protogen was 1.17 *µ*M. The site-directed mutagenesis studies on tobacco *mt*PPO revealed a range of decreased binding affinities of the mutants for protogen: F392H (inactive), F392E (*K*_m_ = 11.2 *µ*M), L356N (*K*_m_ = 11.0 *µ*M), L356V (*K*_m_ = 7.3 *µ*M), L372N (*K*_m_ = 16.4 *µ*M), L372V (*K*_m_ = 103 *µ*M), R98K (*K*_m_ = 2.6 *µ*M), R98E (*K*_m_ = 12.5 *µ*M), and R98A (*K*_m_ = 8.3 *µ*M). The binding free energy shifts of protogen with mutants was also estimated (**Table 2**). Plots of the experimental vs. the calculated values showed a good linear relationship with a correlation coefficient of *r* ^2^ = 0.95 further confirming the reliability of the substrate binding model in tobacco *mt*PPO (The binding models of each mutant are shown in **Figure S5**).

**Table 2:** Calculated Binding Free Energy Shifts (kcal/mol) in Comparison with Those Derived from Experimental Data.

*mt*PPO^a^	ΔΔH^b^	−TΔΔS	ΔΔG		*h*PPO	ΔΔH^b^	−TΔΔS	ΔΔG	
			calc.	expt.				calc.^c^	expt.^d^
F392E	5.82	−1.34	4.48	1.34	M368Q	3.62	0.49	4.11	1.13
L356N	3.40	0.65	4.05	1.33	M368K	1.98	1.04	3.02	0.59
L356V	4.32	−0.48	3.84	1.09	R168S	3.66	0.01	3.67	1.01
L372N	5.97	−1.38	4.59	1.57	G169A	1.00	−0.80	0.20	0.02
L372V	6.64	0.62	7.26	2.66	V170T	−0.61	1.09	0.48	0.21
R98K	3.76	−2.45	1.31	0.47	L166N	1.15	1.04	2.19	0.54
R98E	5.26	−0.89	4.37	1.41	R97G	4.96	−0.22	4.74	1.33
R98A	6.83	−3.56	3.28	1.16	F331T	0.13	0.41	0.54	0.22
					F331A	1.57	1.06	2.63	0.69
					L334V	1.87	1.25	3.12	0.73

The data were determined using both tobacco *mt*PPO and *h*PPO wild type proteins and the indicated mutants.

[a] The kinetic data of tobacco PPO mutants were reported in ref. 20.

[b] ΔΔ = Δ (MT) -Δ (WT).

[c] ΔΔG_calt._ were calculated by Computational Mutation Scanning (CMS) protocol based on the WT binding model (see ref. 42).

[d] ΔΔG_expt._ (kcal/mol) = −RT ln(K_mMut_/K_mWT_), where R is the ideal gas constant, and T is the temperature in K.

Theoretically, PPO from different species with similar folding and substrate binding envelopes should have similar substrate binding models. Previously, we have determined the crystal structure of human PPO (*h*PPO) at a resolution of 1.9 Å and found that it has very similar three dimensional structure and conserved substrate binding envelope as compared to that of tobacco *mt*PPO [21], [22]. Hence, we constructed the three dimensional structure of protogen-bound *h*PPO by superimposing it with the tobacco *mt*PPO-substrate complex and we performed subsequent energy minimization (**Figure 2B**). According to the sequence alignment and structure superimposition between tobacco *mt*PPO and *h*PPO, residues R97, L166, R168, G169, V170, F331, L334, and M368 of *h*PPO correspond to the respective residues R98, F172, A174, G175, T176, F353, L356, and F392 of tobacco *mt*PPO. The binding model revealed that for *h*PPO, ring A of protogen interacted with R168 and was kept in position by hydrophobic interaction with M368. Ring B of protogen is sandwiched between *h*PPO residue V170 and FAD. Residues involved in the interaction with ring C of protogen were L166 and G169 and ring D was sandwiched between *h*PPO residues F331 and L334. The propionate groups of rings C and D could form hydrogen bonds with R97.

In order to further validate the substrate binding model of PPO, site-directed mutagenesis and enzyme kinetic studies were performed on *h*PPO. We constructed an appropriate set of mutant *h*PPO genes, isolated the respective mutant proteins and determined their kinetic parameters *via* continuous fluorometric method and compared the results with wild-type *h*PPO. We introduced both conservative and non-conservative amino acid exchanges for residues of interest in order to evaluate their chemical contribution to the binding of substrate and catalysis. The apparent *K*_m_ value of purified wild-type *h*PPO for protogen was 2.08 *µ*M with a *k*_cat_ value of 2.97 min^−1^ (**Table 3**). The protogen binding model was evaluated by a series of mutagenesis on *h*PPO (**Table 3**). Substitution of Ser for R168 resulted in a 5.4-fold loss of binding for protogen. When M368 was mutated to glutamine, the *K*_m_ for protogen declined by 6.7-fold, while introduction of lysine at the same site decreased protogen binding by 2.7-fold, compared to wild type *h*PPO. To mimic the corresponding residue in tobacco *mt*PPO, V170 was mutated to threonine. V170T was reasonably active, with an acceptable catalytic efficiency and only a slight decrease of protogen binding activity. For the mutants L166N and G169A, protogen binding was reduced by a modest 2.5-fold or was virtually unaffected. However, for these two mutants the reduced catalytic efficiency (*K*_m_/*k*_cat_ = 0.35 *µ*Μ^−1^ • min^−1^ and *K*_m_/*k*_cat_ = 0.17 *µ*Μ^−1^ • min^−1^ respectively), demonstrating their important roles for *h*PPO catalysis. Consistently, the mutants R97G, F331T, F331A, and L334V showed decreased binding capacity (*K*_m_ = 19.64 *µ*M, 3.02 *µ*M, 6.60 *µ*M, and 7.08 *µ*M respectively) compared with the wild-type. The binding free energy shifts of protogen with mutants were also estimated (**Table 2**). Plots of the experimental values vs. the calculated values were linear, with a correlation coefficient of *r* ^2^ = 0.95 further quantitatively confirming the reliability of the substrate binding model in *h*PPO (The binding models of each mutant are shown in **Figure S6**).

**Table 3:** Kinetic parameters of wild-type and mutant *h*PPO.

Protein mutants	*K*_m_ (*µ*Μ)	*k*_cat_ (min^−1^)	*k*_cat_/*K*_m_(*µ*Μ^−1^ • min^−1^)
Wild-type (WT)	2.08±0.11	2.97±0.15	1.43
Substrate ring A			
R168S	11.33±0.93	3.32±0.22	0.29
M368Q	13.89±1.29	9.29±0.75	0.67
M368K	5.63±0.26	14.17±1.10	2.52
Substrate ring B			
V170T	2.97±0.31	7.70±0.66	2.59
Substrate ring C			
L166N	5.13±0.71	1.82±0.13	0.35
G169A	2.16±0.17	0.37±0.04	0.17
Substrate ring D			
R97G	19.64±2.14	76.37±9.15	3.89
F331T	3.02±0.28	9.08±0.76	3.01
F331A	6.60±0.72	4.36±0.39	0.66
L334V	7.08±0.54	51.19±6.21	7.23

The determination of kinetic constants was performed as described in the Experimental section. The Michaelis–Menten constant *K*_m_, the *k*_cat_ and the *k*_cat_/*K*_m_ values for *h*PPO were determined from substrate velocity plots by measuring the constant velocity formation of proto from protogen.

By comprehensively integrating HBE, binding free energy, conformational energy penalty, reaction orientation and coordinates, and with the aid of mutagenesis studies, the model for the binding of protogen to PPO was identified (**Figure 2**). Consequently, the binding model of the product of the reaction, proto, can also be identified (**Figure S7**).

### Binding Pathway and Free Energy Profile

**Figure 3:**
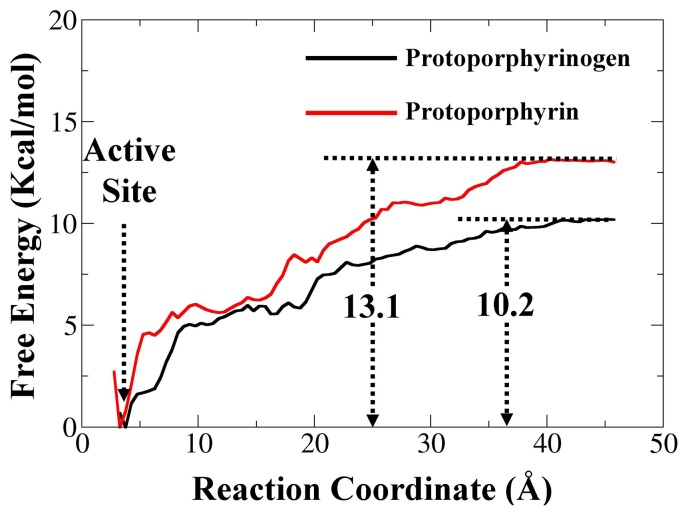
Free energy profiles determined for PPO binding with protogen (black curve) and proto (red curve). The reaction coordinate was defined as the distance between the methylene bridge atom in the *meso* position of protogen and the N5 atom of the FAD. The binding free energy corresponding to protogen and proto of the transformation process are also labeled (units of kcal/mol).

Conventional MD does not clearly simulate the processes of binding and dissociation of substrate and product at the atomic level and therefore is not useful for investigating the protogen/proto recognition by PPO. Therefore, we undertook potential of mean force (PMF) simulations based on the states of protogen- and proto-bound PPOs. During the PMF simulations, the reaction coordinate (RC) is defined as the distance from the mass center of the non-hydrogen atoms of protogen/proto to the mass center of the non-hydrogen atoms of PPO, which was determined based on the structural features. To better understand the energy barriers of the transition, a free energy profile of the entire process was needed. So, based on the data collected from PMF simulations, the corresponding free energy profiles of the egress processes of protogen and proto were determined (**Figure 3**).

**Figure 4:**
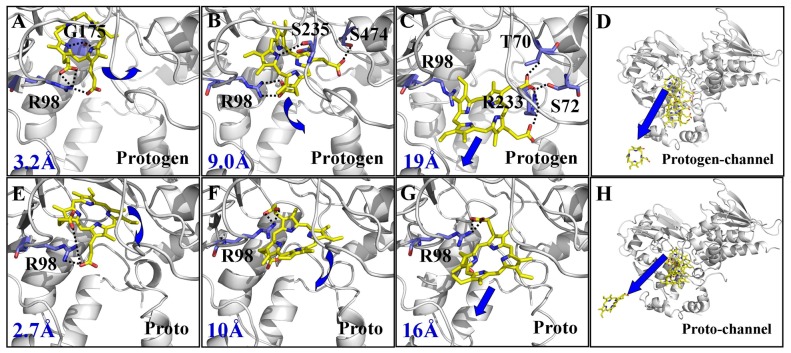
The intermediate states of protogen/proto in the egress process derived from PMF simulations. **A**, Binding structure of protogen in the active site derived from the snapshot at the beginning of PMF trajectory. The carboxyl oxygen atoms of protogen can form three hydrogen bonds with the amino NH hydrogen atoms of R98 and the nitrogen atoms of the pyrrole can form two hydrogen bonds with G175. Tobacco *mt*PPO is displayed as white ribbon. Protogen and its recognition residues are displayed in stick style. Residues of tobacco *mt*PPO are colored by blue and protogen is colored in yellow. Dashed lines represent the hydrogen bond distances. **B**, View of the counterclockwise rotation of protogen in the egress process with RC = ∼9.0 Å. With the counterbalance of interactions switched, one of the carboxyl oxygen atoms of protogen can form new contacts with S474 and one of the nitrogen atoms of the pyrrole can hydrogen bond with S235. **C**, The complex structure of the transition state with RC = ∼19.0 Å. Protogen passes through the opening with the carboxyl oxygen atoms interacting with T70, S72, and R233. **D**, Side view of protogen passing through the new channel. **E**, Binding structure of proto in the active site derived from the snapshot at the beginning of PMF trajectory. The carboxyl oxygen atoms of proto can form three hydrogen bonds with the amino NH hydrogen atoms of R98. **F**, View of the clockwise rotation of proto in the egress process, when RC = ∼10.0 Å with maintenance of the hydrogen bonding between the carboxyl oxygen atoms of proto and the amino NH hydrogen atoms of R98, which was favorable for proto passing through the channel. **G**, The complex structure of the transition state with RC = ∼16.0 Å. Proto passing through the opening with the carboxyl oxygen atoms maintaining interaction with R98. **H**. Side view of proto passing through the new channel.

Along the chosen RC, no energy barrier was identified from the free energy profile of the two egress processes. For the substrate protogen, the minimum of the free energy curve was stabilized with RC = ∼3.2 Å, corresponding to the event when the carboxyl oxygen atoms of protogen formed three hydrogen bonds with R98 in tobacco *mt*PPO, and when the nitrogen atoms of the pyrrole formed two hydrogen bonds with G175 (**Figure 4A**). With the increase of the free energy profile, the protogen began to detach from the binding site with the breakup of the initial interactions. Two plateaus in the free energy profile were observed from ∼5.0 Å to 6.5 Å and ∼9.0 to 11.0 Å of the RC, reflecting the counterbalance of interactions that switched with a counterclockwise rotation around the binding pocket. For example, the carboxyl oxygen atoms of the side chain formed new contacts with S474, while the nitrogen atoms of the pyrrole formed new contacts with S235, when RC = ∼9.0 Å (**Figure 4B**). After slowly increasing from ∼11.0 to 14.5 Å of RC, a fluctuation of the free energy profile was observed from ∼14.5 to 20.0 Å of RC and several energy wells occurred, corresponding to the local binding event on the entrance of the pocket. For example, the carboxyl oxygen atoms of protogen formed new contacts with T70, S72, and R233 with RC = ∼19.0 Å (**Figure 4C**). Finally, a slow increase in the curve occurred from ∼20.0 to 40.0 Å of RC as the protogen pulled away (**Figure 4D**) and converged at 10.2 kcal/mol (**Figure 3**).

In contrast, the outcome for the simulated structure of the product proto was remarkably different. No plateau in the free energy profile was observed from the active site to the outside and a clockwise rotation of proto was observed. The minimum of the free energy curve stabilized at RC = ∼2.7 Å, corresponding to the event when the carboxyl oxygen atoms of proto formed three hydrogen bonds with the amino nitrogen atoms of R98 (**Figure 4E**). The curve increased steeply from ∼3.0 to 5.0 Å followed by a fluctuating rise from ∼5.0 to 10.0 Å of RC with a small energy barrier appearing at RC = ∼10.0 Å, which corresponded to the event of the breaking of the initial interactions. One of the hydrogen bonds between the carbonyl oxygen atom of the propionate group and the amino nitrogen atom of R98 was destroyed with the clockwise rotation of proto around the entrance of the binding pocket (**Figure 4F**). Then the free energy profile was slowly increased from ∼10.0 to 16.0 Å of the RC, which could also be reflected by maintaining one of the hydrogen bonds between the carbonyl oxygen atom of the propionate group and the amino nitrogen atom of R98 (**Figure 4G**). After that, the curve slowly increased as the proto left the active site (**Figure 4H**) and finally converged at 13.1 kcal/mol (**Figure 3**). According to the PMF simulated transient intermediates of protogen and proto, the same residue, R98, formed hydrogen bond interactions throughout the recognition process. The participation of R98 was more important for proto than for protogen in the recognition process. From the PMF calculation, we deduced that the protogen binding process should be very fast, but the proto dissociation process should be slower. To explore the possible role of R98 during the recognition mechanism, the structures of WT and R98A PPO were compared (**Figure S8**). R98 was located in the pocket between the active site and the pathway. Hence, the replacement of R98 with the non-polar amino acid alanine could enlarge the mouth of the pocket and improve the turnover of protogen/proto in the absence of the hydrogen bond, which was especially more favorable to establish an orientation for the proto egress and thus improve the enzyme activity. A previous study established that the R98A mutant had an ∼8-fold larger Michaelis-Menten constant (*K*_m_ = 1.17 *µ*M in WT and = 8.30 *µ*M in R98A) and a ∼60-fold (*k*_cat_ = 6 s^−1^ in WT and = 365 s^−1 ^in R98A) improved enzyme activity [20]. This result further supported the recognition mechanism discovered here and suggested that R98 was more important for proto than protogen in the recognition process.

To further examine the accuracy of the values of binding free energies (ΔG_bind_) as calculated by PMF, the corresponding experimental values of ΔG_bind_ for protogen were also estimated from the available experimental data (*i.e.*, the experimental values of *K*_m_) using the well-known rapid equilibration assumption, *i.e.*, *K*_m_ ≈ *K*_d_ (dissociation constant) [23]. Tobacco *mt*PPO exhibited a value of *K*_m_ = 1.17 *µ*M for protogen [20]. Based on this assumption, we may calculate that Δ*G*_bind,expt._ = −RTln*K*_d_ ≈ -RTln*K*_m_ = −8.1 kcal/mol for PPO binding with protogen. The finally estimated ΔG_bind,calt._ = −10.2 kcal/mol of protogen compares well with Δ*G*_bind,expt._ = −8.1 kcal/mol, although the PMF calculation overestimated the binding affinity by ∼2 kcal/mol, which was likely due to the finite size of the simulation box and the inherent limits of the empirical force field.

### Enzyme Kinetics

**Figure 5:**
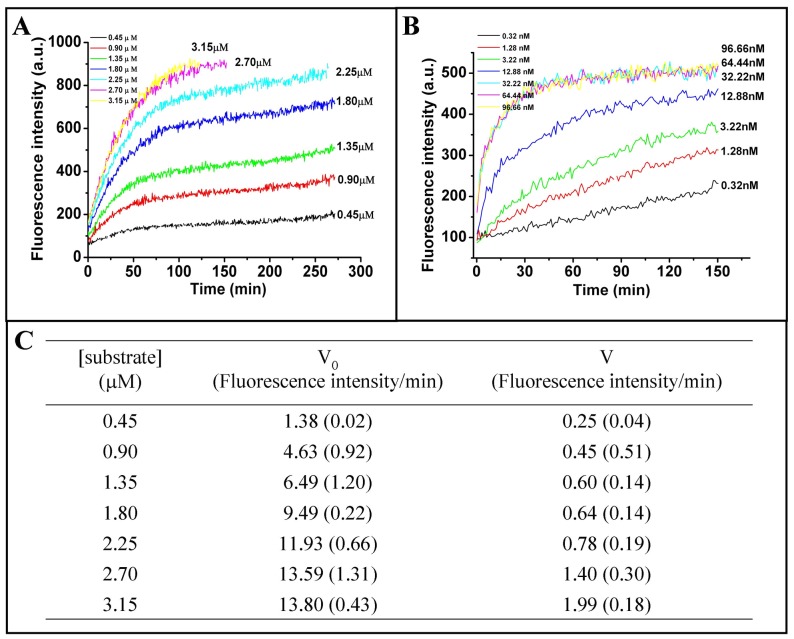
A comparison of the conversion of protoporphyrinogen IX to protoporphyrin IX as monitored by fluorescence assay as catalyzed by PPO. **A**, The enzyme kinetic time-courses with increasing protogen concentrations. The auto-oxidation time-course was excluded from the curve. Reactions were initiated by the addition of enzyme. Data were obtained in the presence of the indicated concentrations of protogen. **B**, Kinetics of the enzymatic catalysis of a fixed amount of protogen (0.34 *µ*M) with different concentrations of *h*PPO. Data were obtained in the presence of the indicated concentrations of *h*PPO enzyme. **C**. The initial velocity (V_0_) and velocity after inflexion (V) (steady state) of each curve in **Figure 5A**. The values in the parentheses are the standard deviations.

From PMF-derived values for ΔG_bind_ (PPO-protogen) and ΔG_bind_ (PPO-proto) and the MM/PBSA results of protogen and proto (**Table S2**), proto should have a stronger binding affinity than protogen and should regulate PPO by feedback-inhibition. Kinetic studies of the enzyme-catalytic oxidation activity were determined *via* a continuous fluorescence method and were examined in conjunction with the data from the auto-oxidation of protogen in order to examine the occurrence of feedback inhibition. The initial phase of product formation curves was linear, but decreased with time, approaching straight lines (steady states) (**Figure 5A**). However, the product formation was not complete (see the velocities in **Figure 5C**). This kind of kinetic time-course demonstrated that the enzymatic activity decreased gradually along with the product formation and finally the enzyme became inhibited. The curvilinear functions displayed by the curves were consistent with the presence of a slow, tight-binding inhibitor [24]. This type of kinetic behavior is usually due to a process characterized by the rapid formation of reactant-enzyme complex, followed by a slower dissociation of the product-enzyme complex [25]. The steady states of the product formation curves exhibited a trend of slow rise after the inflection point (**Figure 5A**), which showed that the enzyme was slowly becoming inhibited by the accumulation of product.

To further verify feedback inhibition, different concentrations of PPO enzyme were assayed to catalyze a fixed amount of protogen. The product inhibition phenomenon was obvious at low concentrations of PPO (**Figure 5B**), but as the concentration of PPO increased, the reaction finished within ∼30 min and no product inhibition was observed. Therefore, product inhibition of PPO only existed at limited concentration of enzyme and a certain amount of product accumulation, which is a hallmark of feedback inhibition mechanism [26], [27]. In addition, the occurrence of feedback inhibition in agreement with, in turn, that the computation-derived relative order, *i.e.,* Δ*G*_bind_ (PPO-protogen)>Δ*G*_bind_ (PPO-proto), is reliable.

### Conclusion

In summary, a novel binding model for protogen with PPO has been identified by combining extensive computational simulations and mutagenesis studies. Based on this newly identified binding model, the enzyme-substrate (ES), enzyme-product (EP) recognition pathway, the corresponding free energy profiles, and the feedback inhibition mechanism have been identified. The simulated PMF free energy profile was barrierless for protogen binding, which indicates that the protogen binding process should be very fast. But the free energy profiles showed that the dissociation process for proto should be slower than protogen, indicating that PPO activity is modulated by a feedback inhibition with respect to proto. The kinetic time courses also displayed a burst of product formation followed by a linear steady-state rate when reactions were initiated by the addition of enzyme, which is a hallmark of feedback inhibition. We believed that the established structural and energetic insights into the entire ES binding and EP dissociation process provide a valuable basis for future structure- and product-based design of highly potent PPO inhibitors for application in the development of agrochemicals or PDT cancer therapy.

## Methods

### System Setup and Molecular Docking

The X-ray crystal structure of *Nicotiana tabacum* PPO (*mt*PPO) used for the study was downloaded from RCSB [19]. The initial structure was revised first by means of adding lost residues and hydrogen atoms, checking bonds and bumps, then energy minimized for 2,000 steps of steepest descent calculations and 2,000 steps of conjugated gradient calculations by using SYBYL 7.0 (Tripos Inc., USA). Four kinds of the macrocycle conformations of protogen were constructed by using SYBYL 7.0 (**Figure S1**). The molecular geometries of the four conformations were optimized by performing *ab initio* electronic structure calculation with Gaussian03 program at the HF/6-31+G* level [28]. The optimized geometries were used to construct the entire structures of protogen and the final structures of different conformations were optimized with the macrocycle fixed by using conjugated gradient in SYBYL 7.0. The different conformations were used as the starting structures for docking studies.

Docking calculations were performed on these conformations with AutoDock4.0 [29]. The protein and ligand structures were prepared with AutoDock Tools [30]. The atomic Gasteiger-Huckel charges were assigned to the ligand and receptor. A total of 256 runs were launched. Most of the parameters for the docking calculation were set to the default values recommended by the software. Each docked structure was scored by the built-in scoring function and was clustered by 0.8 Å of RMSD criterions. For each binding model, molecular mechanics/Poisson-Boltzmann surface area (MM/PBSA) was performed (see details in **Table S1**). Before the MM/PBSA calculation, the complex structure was further refined with the steepest descent algorithm first and then the conjugated gradient algorithm by using the AMBER9 package [31]. During the energy minimization process, the receptor was first fixed and only the ligand was kept free; then the ligand and residue sidechains were kept free; finally all atoms of the system were kept free and refined to a convergence of 0.01 kcal/(mol·Å). To avoid the drawbacks of the Autodock program and the conformational analysis method, we also tested other docking programs and methods. See Supporting Information (**Figure S2**) for detailed results.

### Molecular Dynamics Simulation and Free Energy Calculation

Based on the analysis of docking results, two binding models were selected for MD simulation. Prior to that, with the Gaussian-optimized geometries, the electrostatic potential and partial atomic charges were determined by performing the electrostatic potential (ESP) fitting according to the Merz–Singh–Kollman scheme [32], [33]. The RESP charges of the ligand was produced by using the standard RESP protocol [34], [35] implemented in the Antechamber module of the AMBER9 program [31]. The system was solvated in an octahedral box of TIP3P water with the crystallographic water molecules kept. The edge of the box was at least 10 Å from the solute. Appropriate sodium counterions were added to the system to preserve neutrality. The solvated system had a total of about 58,496 atoms, with about 7,381 of them belonging to the solute. Before the MD simulation, some energy minimization steps were applied to the system. First, the solute was kept fixed with a constraint of 500 kcal mol^−1 ^Å^−2^, waters and counterions were minimized; the backbone atoms of the protein were then fixed with the ligand, sidechains, and other atoms free to move; finally, the entire system was fully minimized without any constraint. In each step, energy minimization was first performed by using the steepest descent algorithm for 2,000 steps and then the conjugated gradient algorithm for another 3,000 steps.

The MD simulation was performed under periodic boundary conditions by using the Sander module of the AMBER9 program, as we have done for the same protein before [36]. First, the system was fixed to make the heating only for waters and counterions for 10 ps to make sure the solute was fully solvated; then, the whole system was gradually heated from 10 to 298 K by weak-coupling method [37] and equilibrated for 100 ps with the protein backbone fixed; lastly, the system was switched to a constant pressure equilibration to 3 ns. During the MD simulation, the particle mesh Ewald (PME) algorithm [38], [39] was used to deal with long-range electrostatic interactions with a cutoff distance of 10 Å, which was also used for the van der Waals (vdW) energy terms. All of the angles and bonds involving hydrogen atoms were constrained by using the SHAKE algorithm [40]. The time step used for the MD simulations was 2.0 fs and the coordinates were collected every 1 ps.

Because the flexibility of the side chain and the ligand-protein interaction was of concern, a shorter simulation time (3 ns) was adopted. The MD simulations were performed with all the hydrogen bond distances constrained at the beginning, which were then slowly relaxed. As shown by the small fluctuations of the key distances along the simulation time in **Figure S3**, the MD simulations reached equilibrium after ∼1 ns of simulations. The stable MD trajectory was used to perform the binding free energy (Δ*G*_bind_) calculation by using MM/PBSA method [41]. A total of 1,000 snapshots were taken from the last 1 ns trajectory with an interval of 1 ps to analyze the binding energy and at the same time, the counterions and water molecules (waters related to the crucial hydrogen bond were not included) were stripped. Our MM/PBSA calculation was performed in the same way as we reported elsewhere [42]. In order to evaluate the convergence of the MD simulation, the MM/PBSA calculation with snapshots from different intervals of the last 1 ns trajectory were compared (see details in **Table S2**). Further, the MD simulations of M14 and M15 were also repeated with different sets of parameters and force-field (see details in **Figure S4**). The final binding free energy was determined as the average of all the snapshots and the standard errors were also calculated with the same method reported before [42]. Finally, hydrogen bond energies (HBE) were also calculated by using a previously published method [43].

### Potential of Mean Force (PMF) Calculations

In order to explore the free energy profile of the PPO-protogen/proto binding and dissociation process, PMF calculations were performed by using umbrella-sampling MD simulations [44]. In this calculation the reaction coordinate was divided into 120 windows separated by 0.1, 0.2, and 0.5 Å, each of which is sampled separately. The initial complex structure was adopted directly from our previous MD simulations. The biasing force constant applied in different windows of umbrella-sampling ranged from 4.0 to 60.0 kcal/mol•Å^2^. The selected structure for each window was first equilibrated for 50 ps and then kept running for 1,000 ps. In order to examine the convergence of the PMF results, additional PMF calculations with different intervals were performed (provided as **Figure S9**). The three curves (associated with the MD simulations during 0.2∼0.6, 0.2∼0.8, and 0.2∼1.0 ns) depicted in **Figure S9** are indistinguishable or identical, which suggests 1.0 ns is sufficient for each window of the PMF simulations. Finally, the data collected from separate simulation windows were combined along the reaction coordinate and a 120 ns trajectory was obtained for each system, which were then used to calculate the PMF along the whole recognition pathway with the weighed histogram analysis method (WHAM) [45].

### Kinetic Assays for PPO and its Mutants

The recombinant plasmid (pHPPO-X) was a generous gift from Dr. Harry A. Dailey (University of Georgia, Athens, GA, USA). *h*PPO mutations were generated from the recombinant plasmid (pHPPO-X) using the DpnI-mediated site-directed mutagenesis kit (Biocrest Manufacturing LP, Cedar Creek, TX, USA), and mutations were confirmed by sequence analysis. Expression and purification methods of *h*PPO were the same as used with our previous publication [21].

*h*PPO activity was assayed by measuring the velocity of the formation of proto from protogen on a 96-well plate using the continuous fluorometric method [21]. The product had a maximum excitation wavelength of 410 nm and a maximum emission wavelength of 630 nm. The total volume of the reaction mixture was 200 *µ*L and consisted of 0.1 M potassium phosphate buffer (pH 7.4), 5 mM DTT, 1 mM EDTA, 0.2 M imidazole, and 0.03% Tween 80 (v/v). The reaction was initiated by the addition of substrate, and the autoxidation rate was subtracted. The kinetic parameters, including the Michaelis-Menten constant (*K*_m_), the maximal velocity (V_max_), and the catalytic constant (*k*_cat_), were determined by a Lineweaver-Burk plot.

### Kinetic Assays for Feedback Inhibition

Two experiments were performed to verify the occurrence of feedback inhibition. Experiment 1: The concentration of *h*PPO enzyme was 3.22 nM. The reaction was initiated by the addition of various concentrations of the substrate (0.45, 0.90, 1.35, 1.80, 2.25, 2.70, and 3.15 *µ*M). The auto-oxidation rate of protogen was measured in the absence of enzyme and this rate was excluded from the final enzyme kinetic time-course. Experiment 2: Different concentrations of *h*PPO enzyme (0.32, 1.28, 3.22, 12.88, 32.22, 64.44, and 96.66 nM) were assayed to catalyze a fixed amount of protogen (0.34 *µ*M).

## Supporting Information

Figure S1 The scheme of the spacial conformation of the macrocycle of the substrate.
(TIF)

Figure S2 **A.** Comparison between the obtained binding modes through Autodock (M15) and Gold program (NO. 785). **B.** Comparison between the obtained binding modes through Autodock (M14) and Gold program (NO. 644). **C.** Conformational distribution ratio of the 1000 conformers.
(TIF)

Figure S3 View of the binding modes of the substrate in the PPO active site, plots of key distance changes versus simulation time, and substrate-residues interaction spectrums of M14 and M15.
(TIF)

Figure S4 Plots of RMSD and key distance changes versus simulation time in the repeated MD simulations of M14 and M15.
(TIF)

Figure S5 The models of protogen binding with different *mt*PPO mutants.
(TIF)

Figure S6 The models of protogen binding with different *h*PPO mutants.
(TIF)

Figure S7 View of the binding modes of proto in the PPO active site.
(TIF)

Figure S8 The structural comparison between WT and R98A.
(TIF)

Figure S9 PMF-simulated free energy profiles for PPO binding with protogen (**A**) and proto (**B**).
(TIF)

Table S1 Docking Study Results of Different Conformers.
(DOC)

Table S2 Energetic results of MM-PBSA calculation based on MD sampling and the corresponding convergence analysis.
(DOC)

## References

[1] Dayan FE, Dayan EA (2011) Porphyrins: One ring in the colors of life. Am Sci 99: 236–243.

[2] Poulson R, Polglase WJ (1975) The enzymic conversion of protoporphyrinogen IX to protoporphyrin IX. Protoporphyrinogen oxidase activity in mitochondrial extracts of Saccharomyces cerevisiae. J Biol Chem 250: 1269–1274. 234450

[3] Poulson R (1976) The enzymic conversion of protoporphyrinogen IX to protoporphyrin IX in mammalian mitochondria. J Biol Chem 251: 3730–3733. 6461

[4] Patzoldt WL, Hager AG, McCormick JS, Tranel PJ (2006) A codon deletion confers resistance to herbicides inhibiting protoporphyrinogen oxidase. Proc Natl Acad Sci U S A 103: 12329–12334. 16894159 10.1073/pnas.0603137103 PMC1567880

[5] Kato K, Tanaka R, Sano S, Tanaka A, Hosaka H (2010) Identification of a gene essential for protoporphyrinogen IX oxidase activity in the cyanobacterium Synechocystis sp. PCC6803. Proc Natl Acad Sci U S A 107: 16649–16654. 20823222 10.1073/pnas.1000771107 PMC2944763

[6] Jeong E, Houn T, Kuk Y, Kim ES, Chandru HK, et al (2003) A point mutation of valine-311 to methionine in Bacillus subtilis protoporphyrinogen oxidase does not greatly increase resistance to the diphenyl ether herbicide oxyfluorfen. Bioorg Chem 31: 389–397. 12941291 10.1016/s0045-2068(03)00093-2

[7] Maneli MH, Corrigall AV, Klump HH, Davids LM, Kirsch RE, et al (2003) Kinetic and physical characterisation of recombinant wild-type and mutant human protoporphyrinogen oxidases. Biochim Biophys Acta 1650: 10–21. 12922165 10.1016/s1570-9639(03)00186-9

[8] Dayan FE, Daga PR, Duke SO, Lee RM, Tranel PJ, et al (2010) Biochemical and structural consequences of a glycine deletion in the alpha-8 helix of protoporphyrinogen oxidase. Biochim Biophys Acta 1804: 1548–1556. 20399914 10.1016/j.bbapap.2010.04.004

[9] Arnould S, Camadro JM (1998) The domain structure of protoporphyrinogen oxidase, the molecular target of diphenyl ether-type herbicides. Proc Natl Acad Sci U S A 95: 10553–10558. 9724741 10.1073/pnas.95.18.10553 PMC27932

[10] Matringe M, Camadro JM, Labbe P, Scalla R (1989) Protoporphyrinogen oxidase as a molecular target for diphenyl ether herbicides. Biochem J 260: 231–235. 2775186 10.1042/bj2600231 PMC1138650

[11] Meazza G, Bettarini F, La Porta P, Piccardi P, Signorini E, et al (2004) Synthesis and herbicidal activity of novel heterocyclic protoporphyrinogen oxidase inhibitors. Pest Manag Sci 60: 1178–1188. 15578598 10.1002/ps.923

[12] Volker A, Burkhard G (2005) Antimicrobial photodynamic therapy compound and method of use. US Patent 20050049228.

[13] Moghissi K, Dixon K, Stringer M, Thorpe JA (2009) Photofrin PDT for early stage oesophageal cancer: long term results in 40 patients and literature review. Photodiagnosis Photodyn Ther 6: 159–166. 19932447 10.1016/j.pdpdt.2009.07.026

[14] Robertson CA, Evans DH, Abrahamse H (2009) Photodynamic therapy (PDT): a short review on cellular mechanisms and cancer research applications for PDT. J Photochem Photobiol B 96: 1–8. 19406659 10.1016/j.jphotobiol.2009.04.001

[15] Halling BP, Yuhas DA, Fingar VF, Winkelmann JW (1994) Protoporphyrinogen Oxidase Inhibitors for Tumor Therapy. Porphyric Pesticides: American Chemical Society. 280–290.

[16] Zhang D, Gullingsrud J, McCammon JA (2006) Potentials of mean force for acetylcholine unbinding from the alpha7 nicotinic acetylcholine receptor ligand-binding domain. J Am Chem Soc 128: 3019–3026. 16506783 10.1021/ja057292u PMC2546508

[17] Buch I, Giorgino T, De Fabritiis G (2011) Complete reconstruction of an enzyme-inhibitor binding process by molecular dynamics simulations. Proc Natl Acad Sci U S A 108: 10184–10189. 21646537 10.1073/pnas.1103547108 PMC3121846

[18] Siebel GL, Kollman PA (1990) Comprehensive Medicinal Chemistry. Pergamon, Oxford. 125–138.

[19] Koch M, Breithaupt C, Kiefersauer R, Freigang J, Huber R, et al (2004) Crystal structure of protoporphyrinogen IX oxidase: a key enzyme in haem and chlorophyll biosynthesis. EMBO J 23: 1720–1728. 15057273 10.1038/sj.emboj.7600189 PMC394243

[20] Heinemann IU, Diekmann N, Masoumi A, Koch M, Messerschmidt A, et al (2007) Functional definition of the tobacco protoporphyrinogen IX oxidase substrate-binding site. Biochem J 402: 575–580. 17134376 10.1042/BJ20061321 PMC1863572

[21] Qin X, Tan Y, Wang L, Wang Z, Wang B, et al (2010) Structural insight into human variegate porphyria disease. FASEB J 25: 653–664. 21048046 10.1096/fj.10-170811

[22] Hao GF, Tan Y, Yu NX, Yang GF (2011) Structure-activity relationships of diphenyl-ether as protoporphyrinogen oxidase inhibitors: insights from computational simulations. J Comput Aided Mol Des 25: 213–222. 21259066 10.1007/s10822-011-9412-6

[23] Huang X, Zheng F, Zhan CG (2011) Human butyrylcholinesterase-cocaine binding pathway and free energy profiles by molecular dynamics and potential of mean force simulations. J Phys Chem B 115: 11254–11260. 21902185 10.1021/jp2047807 PMC3179575

[24] Hao GF, Wang F, Li H, Zhu XL, Yang WC, et al (2012) Computational discovery of picomolar Q(o) site inhibitors of cytochrome bc1 complex. J Am Chem Soc 134: 11168–11176. 22690928 10.1021/ja3001908

[25] Frieden E, Walter C (1963) Prevalence and significance of the product inhibition of enzymes. Nature 198: 834–837. 13959750 10.1038/198834a0

[26] Denton H, Fyffe S, Smith TK (2010) GDP-mannose pyrophosphorylase is essential in the bloodstream form of Trypanosoma brucei. Biochem J 425: 603–614. 19919534 10.1042/BJ20090896

[27] Zeldenrust F, Wadman WJ (2009) Two forms of feedback inhibition determine the dynamical state of a small hippocampal network. Neural Netw. 1139–1158. 10.1016/j.neunet.2009.07.015 19679445

[28] Frisch MJ, Trucks GW, Schlegel HB, Scuseria GE, Robb MA, et al.. (2003) Gaussian 03, revision B-03: Gaussian, Inc.: Pittsburgh, PA.

[29] Huey R, Morris GM, Olson AJ, Goodsell DS (2007) A semiempirical free energy force field with charge-based desolvation. J Comput Chem 28: 1145–1152. 17274016 10.1002/jcc.20634

[30] Sanner MF (2005) A component-based software environment for visualizing large macromolecular assemblies. Structure 13: 447–462. 15766546 10.1016/j.str.2005.01.010

[31] Case DA, Darden TA, Cheatham TE, Simmerling CL, Wang J, et al.. (2006) AMBER 9: University of California: San Francisco.

[32] Singh UC, Kollman PA (1984) An approach to computing electrostatic charges for molecules. J Comput Chem 5: 129–145.

[33] Besler BH, Merz KM, Kollman PA (1990) Atomic charges derived from semiempirical methods. J Comput Chem 11: 431–439.

[34] Bayly CI, Cieplak P, Cornell WD, Kollman PA (1993) A well-behaved electrostatic potential based method using charge restraints for deriving atomic charges: the RESP model. J Phys Chem 97: 10269–10280.

[35] Cornell WD, Cieplak P, Bayly CI, Kollman PA (1993) Application of RESP charges to calculate conformational energies, hydrogen bond energies, and free energies of solvation. J Am Chem Soc 115: 9620–9631.

[36] Hao GF, Zhu XL, Ji FQ, Zhang L, Yang GF, et al (2009) Understanding the mechanism of drug resistance due to a codon deletion in protoporphyrinogen oxidase through computational modeling. J Phys Chem B 113: 4865–4875. 19284797 10.1021/jp807442n

[37] Morishita T (2000) Fluctuation formulas in molecular-dynamics simulations with the weak coupling heat bath. J Chem Phys 113: 2976–2982.

[38] Darden T, York D, Pedersen L (1993) Particle mesh Ewald: An N. log(N) method for Ewald sums in large systems. J Chem Phys 98: 10089–10092.

[39] Essmann U, Perera L, Berkowitz ML (1995) A smooth particle mesh Ewald method. J Chem Phys 103: 8577–8593.

[40] Ryckaert JP, Ciccotti G, Berendsen HJC (1977) Numerical integration of the Cartesian equations of mot ion of a system with constraints: molecular dynamics of n-alkanes. J Comput Phys 23: 327–341.

[41] Kollman PA, Massova I, Reyes C, Kuhn B, Huo S, et al (2000) Calculating structures and free energies of complex molecules: combining molecular mechanics and continuum models. Acc Chem Res 33: 889–897. 11123888 10.1021/ar000033j

[42] Hao GF, Yang GF, Zhan CG (2010) Computational mutation scanning and drug resistance mechanisms of HIV-1 protease inhibitors. J Phys Chem B 114: 9663–9676. 20604558 10.1021/jp102546s PMC2916083

[43] Hao GF, Yang GF (2010) The role of Phe82 and Phe351 in auxin-induced substrate perception by TIR1 ubiquitin ligase: a novel insight from molecular dynamics simulations. PLoS One 5: e10742. 20505777 10.1371/journal.pone.0010742 PMC2873998

[44] Torrie GM, Valleau JP (1977) Nonphysical sampling distribution in Monte Carlo free-energy estimation: umbrella sampling. J Comput Phys 23: 187–199.

[45] Kumar S, Bouzida D, Swendsen RH, Kollman PA, Rosenberg J (1992) The weighted histogram analysis method for free-energy calculations on biomolecules. I: The method. J Comput Chem 13: 1011–1021.

[46] Pan Y, Gao D, Yang W, Cho H, Yang G, et al (2005) Computational redesign of human butyrylcholinesterase for anticocaine medication. Proc Natl Acad Sci U S A 102: 16656–16661. 16275916 10.1073/pnas.0507332102 PMC1283827

[47] Gao D, Cho H, Yang W, Pan Y, Yang G, et al (2006) Computational design of a human butyrylcholinesterase mutant for accelerating cocaine hydrolysis based on the transition-state simulation. Angew Chem -Int Edit 45: 653–657. 10.1002/anie.200503025 PMC2878656 16355430

[48] Pan Y, Gao D, Zhan CG (2008) Modeling the catalysis of anti-cocaine catalytic antibody: competing reaction pathways and free energy barriers. J Am Chem Soc 130: 5140–5149. 18341277 10.1021/ja077972s PMC2878667

