# Supplementary figures and images for: Computational and Experimental Insights into the Mechanism of Substrate Recognition and Feedback Inhibition of Protoporphyrinogen Oxidase
Source: PLoS One. 2013 Jul 23;8(7):e69198. doi: 10.1371/journal.pone.0069198 (PMC3720618; doi:10.1371/journal.pone.0069198)

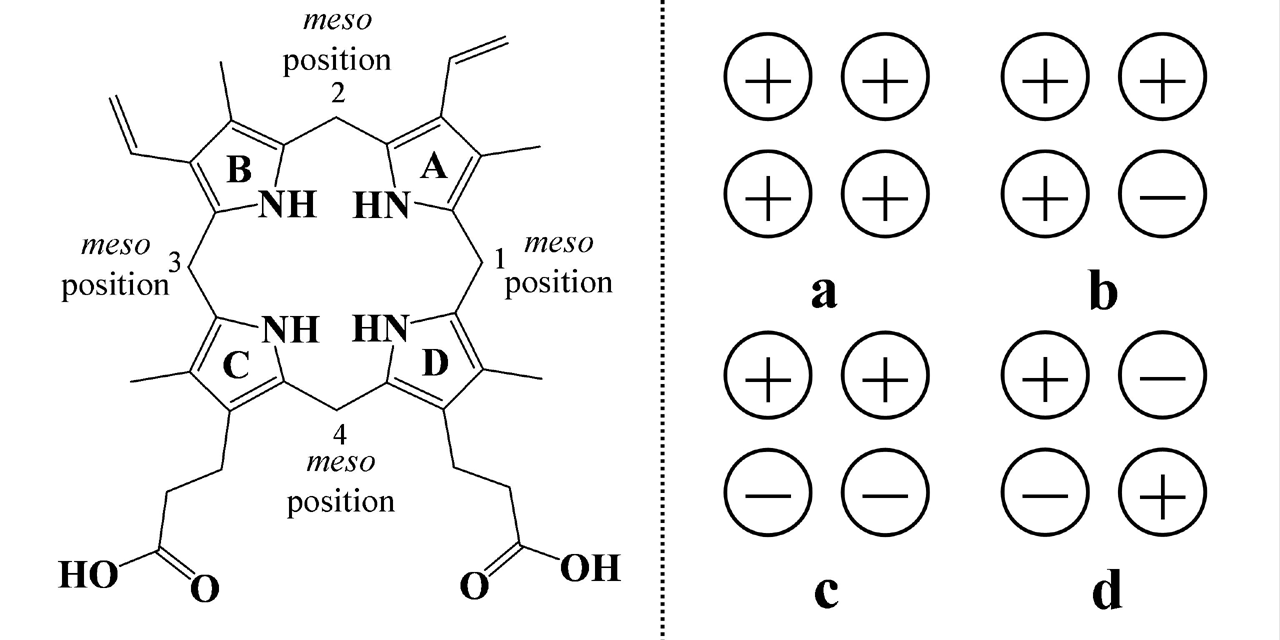

Supplement: Figure S1 — The scheme of the spacial conformation of the macrocycle of the substrate. (TIF) [file pone.0069198.s001.tif]

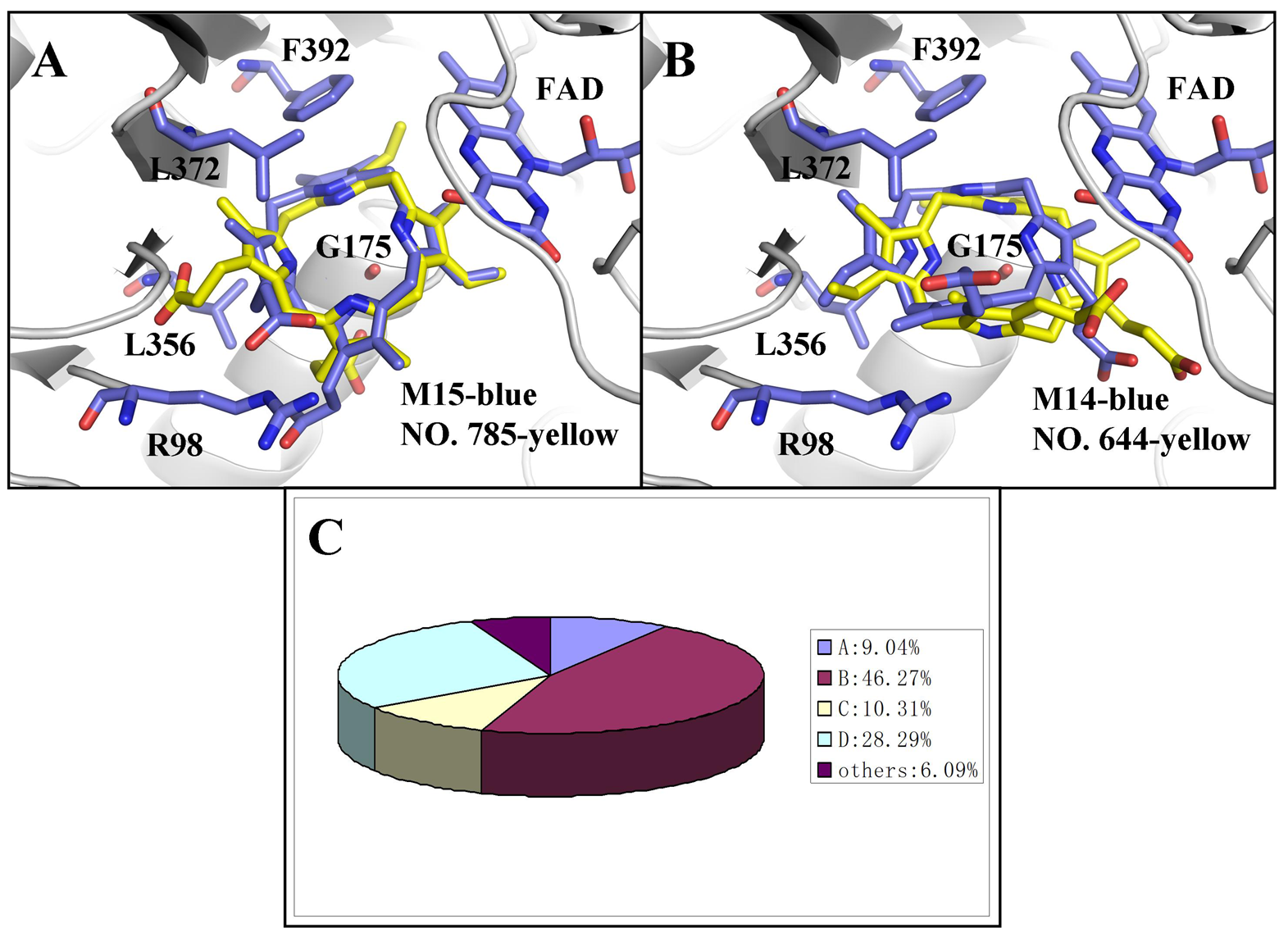

Supplement: Figure S2 — A. Comparison between the obtained binding modes through Autodock (M15) and Gold program (NO. 785). B. Comparison between the obtained binding modes through Autodock (M14) and Gold program (NO. 644). C. Conformational distribution ratio of the 1000 conformers. (TIF) [file pone.0069198.s002.tif]

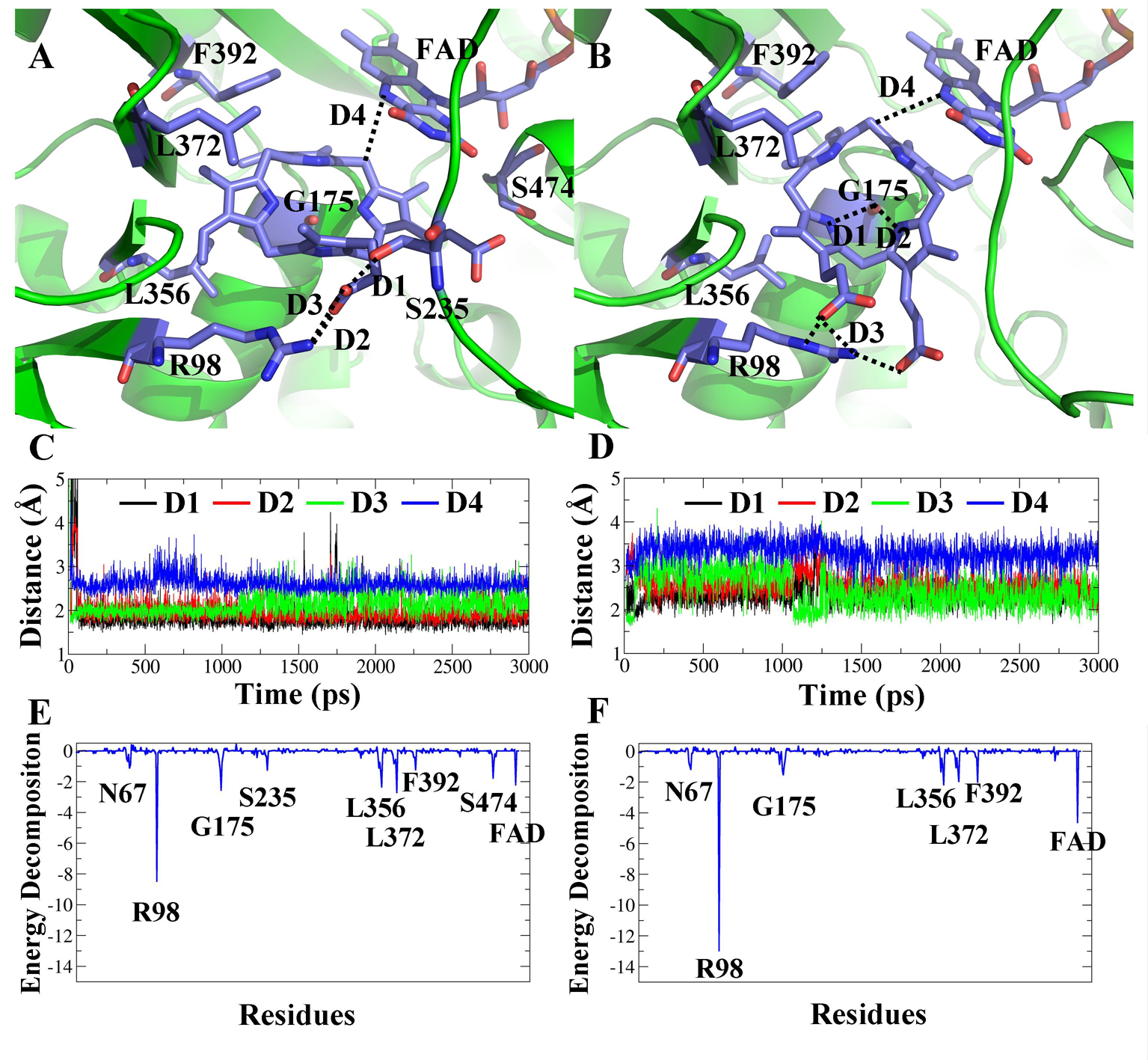

Supplement: Figure S3 — View of the binding modes of the substrate in the PPO active site, plots of key distance changes versus simulation time, and substrate-residues interaction spectrums of M14 and M15. (TIF) [file pone.0069198.s003.tif]

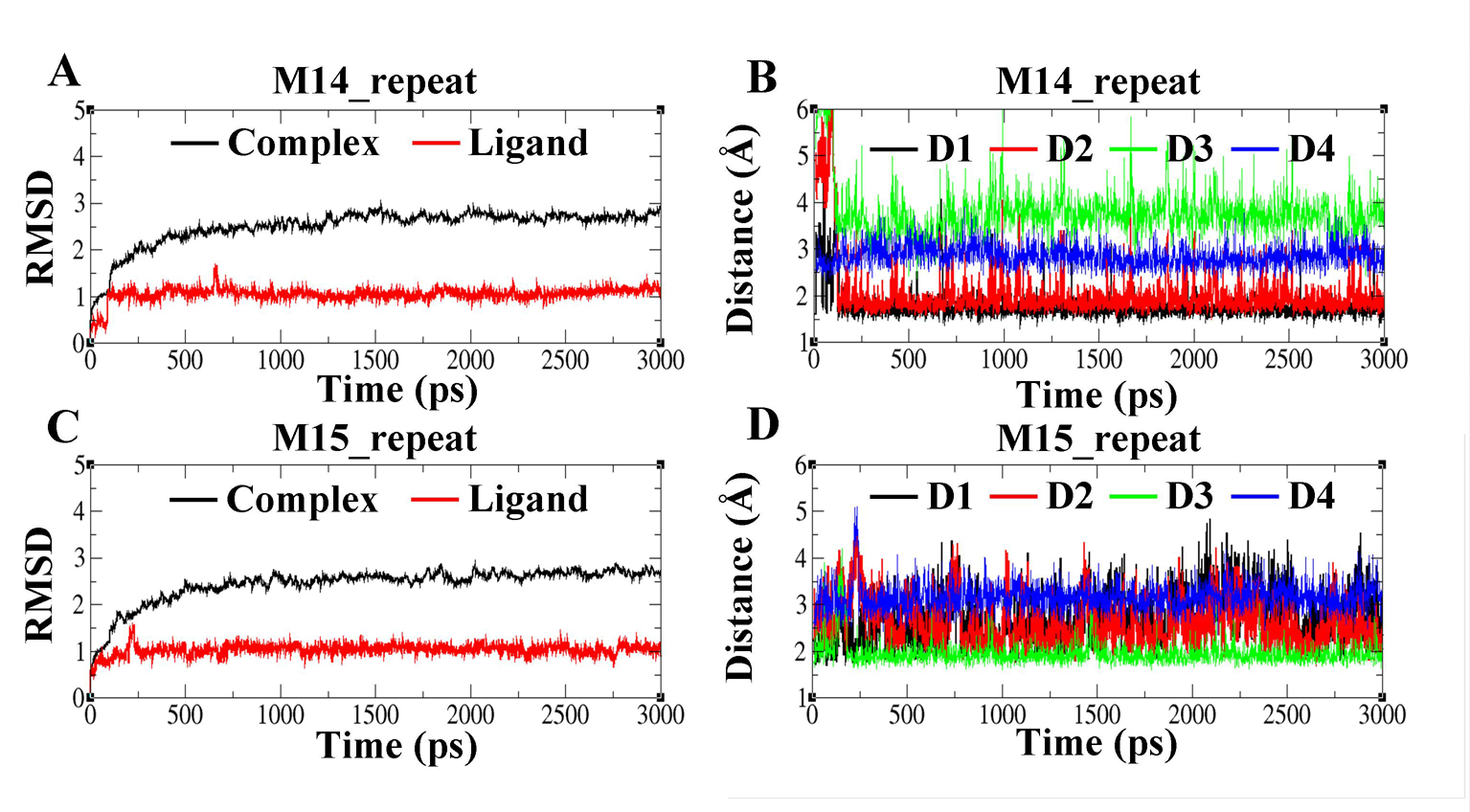

Supplement: Figure S4 — Plots of RMSD and key distance changes versus simulation time in the repeated MD simulations of M14 and M15. (TIF) [file pone.0069198.s004.tif]

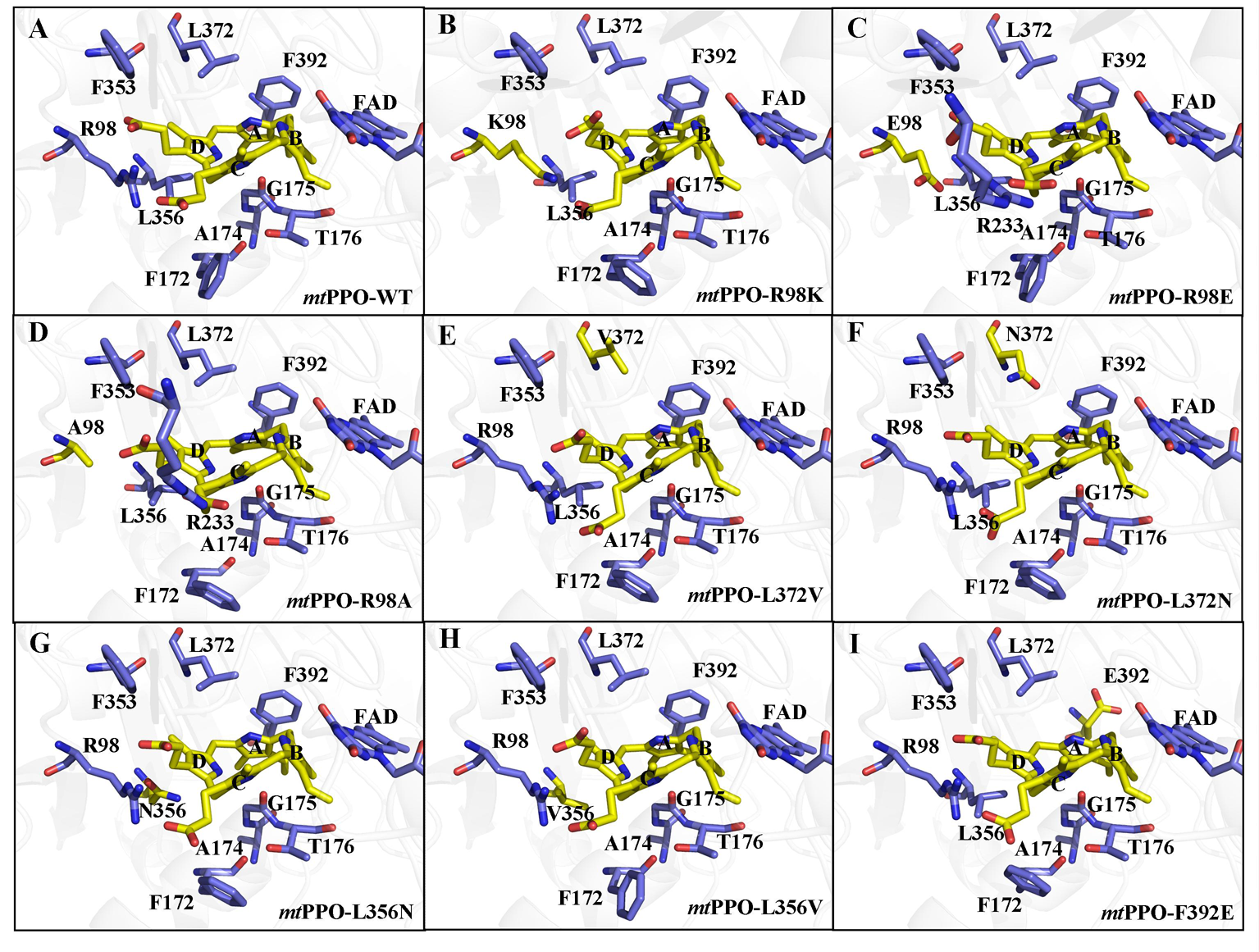

Supplement: Figure S5 — The models of protogen binding with different mtPPO mutants. (TIF) [file pone.0069198.s005.tif]

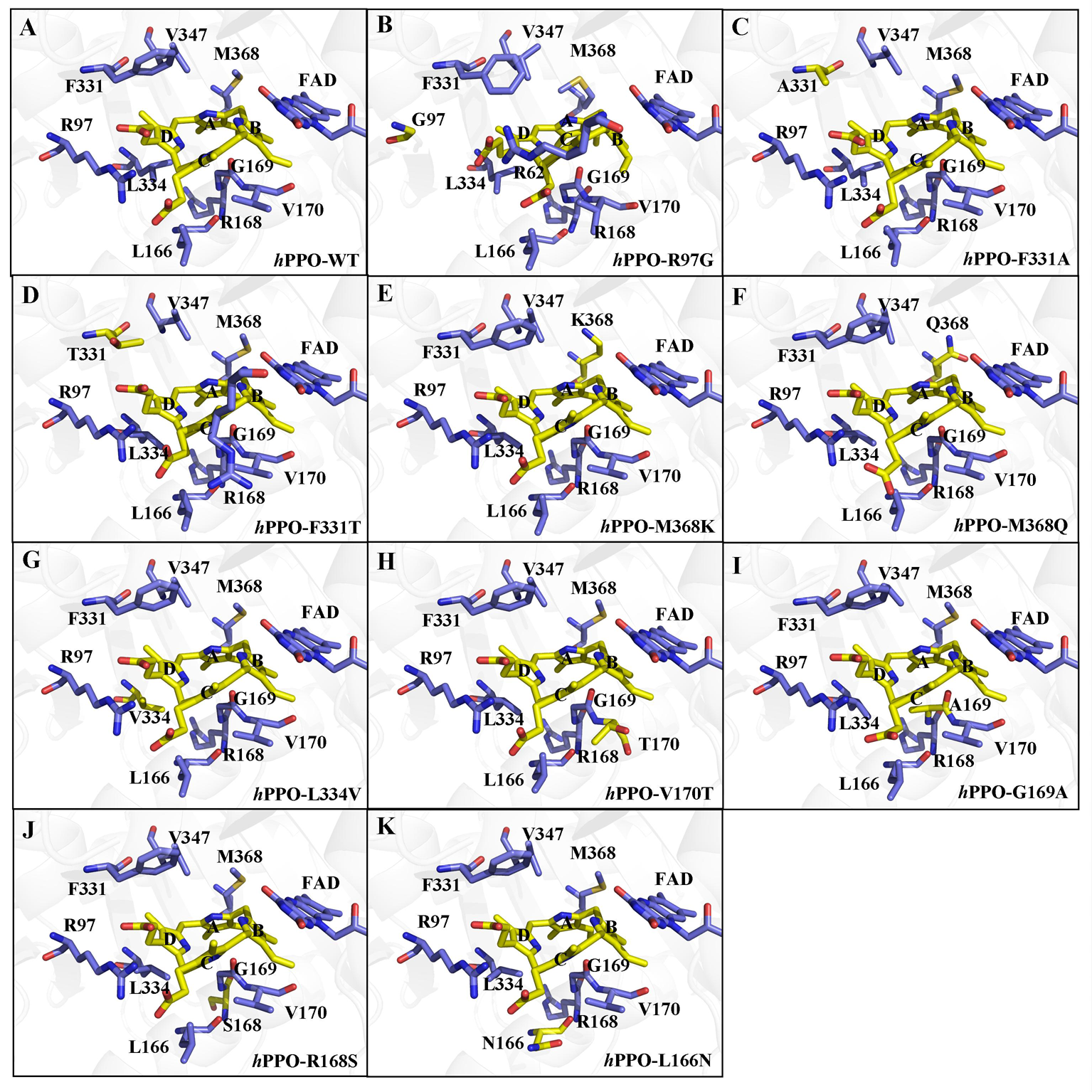

Supplement: Figure S6 — The models of protogen binding with different hPPO mutants. (TIF) [file pone.0069198.s006.tif]

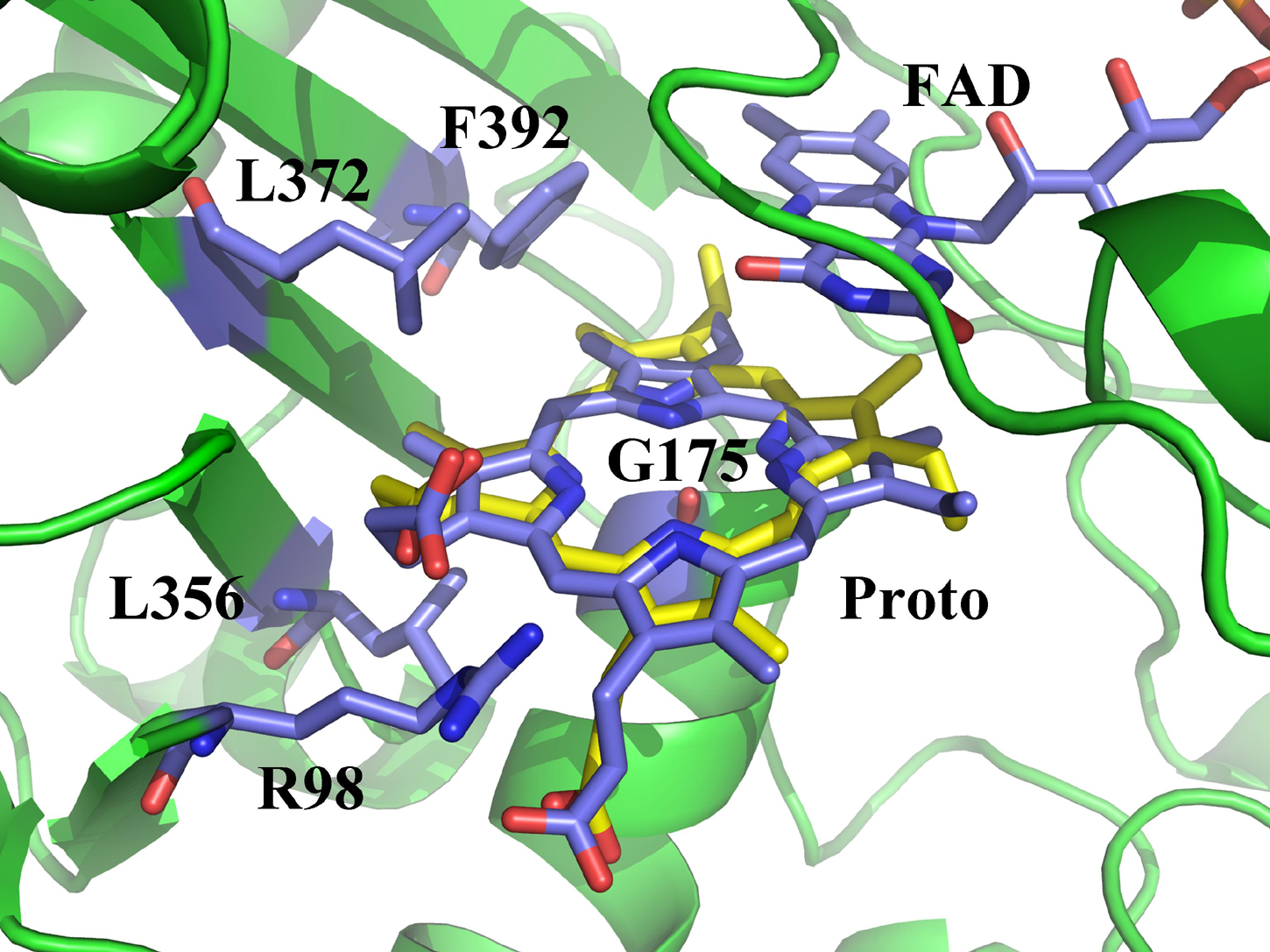

Supplement: Figure S7 — View of the binding modes of proto in the PPO active site. (TIF) [file pone.0069198.s007.tif]

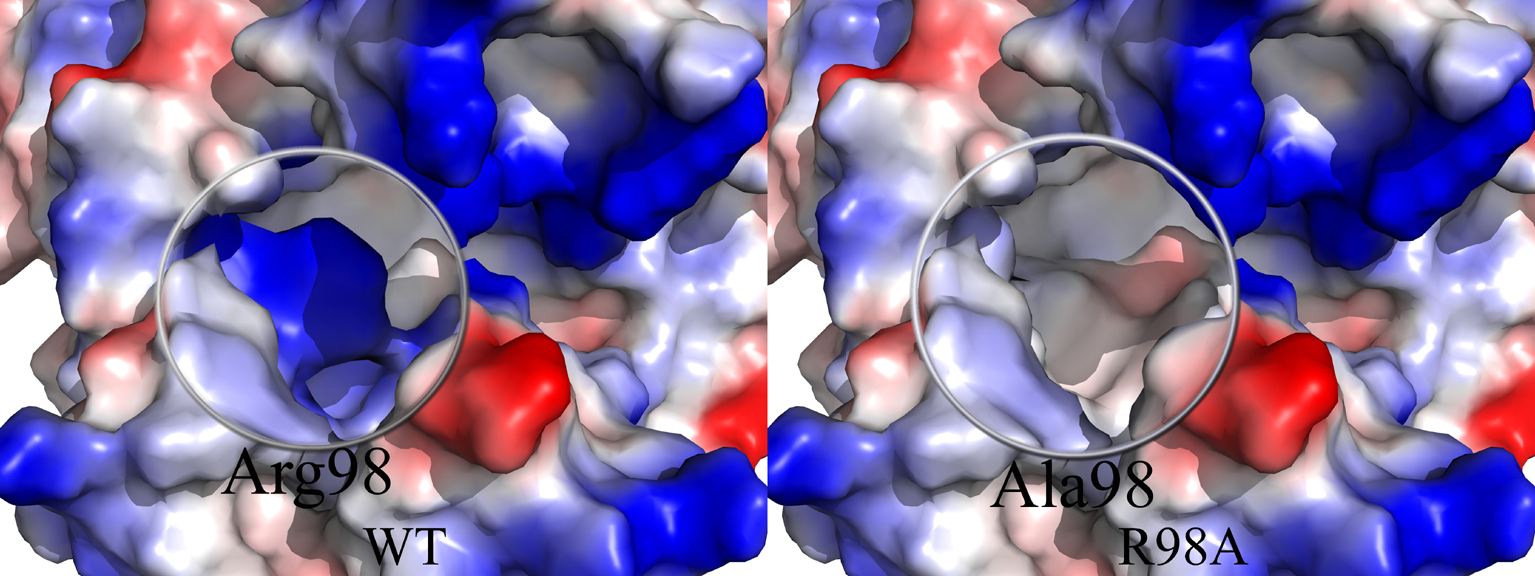

Supplement: Figure S8 — The structural comparison between WT and R98A. (TIF) [file pone.0069198.s008.tif]

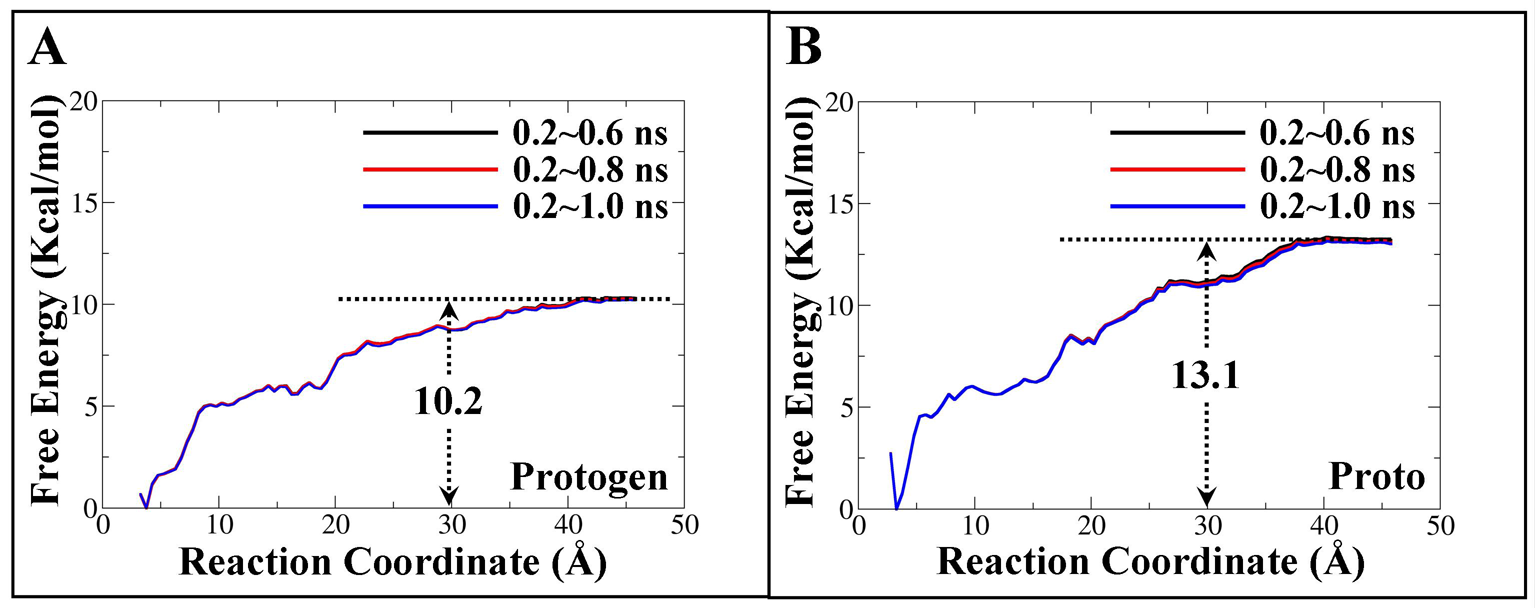

Supplement: Figure S9 — PMF-simulated free energy profiles for PPO binding with protogen (A) and proto (B). (TIF) [file pone.0069198.s009.tif]
