# Supplementary material for: Computational and Experimental Insights into the Mechanism of Substrate Recognition and Feedback Inhibition of Protoporphyrinogen Oxidase
Source: PLoS One. 2013 Jul 23;8(7):e69198. doi: 10.1371/journal.pone.0069198 (PMC3720618; doi:10.1371/journal.pone.0069198)
Supplement: Table S1 — Docking Study Results of Different Conformers. (DOC) [file pone.0069198.s010.doc]

***Table S1:*** Docking Study Results of Different Conformers. According to the catalytic mechanistic hypotheses proposed by Koch *et al* [2], the reaction center (the methylene bridge atom in *meso* position of protogen in **Fig. S1** and the N_5_ atom of the FAD) should be close to each other in the docking modes. So the binding modes with shorter reaction distance and favorable binding affinity in each conformer were selected as representatives. The reaction distances in the binding modes distributed from 3.05 to 6.79 Å, while the binding affinities determined by Autodock4 distributed from -8.25 to -12.48 kcal/mol. Because in our previous studies [3], we noted that the standard Autodock score generally underestimated the hydrogen-bonding energy. Thus, MM-PBSA calculation for the enthalpy contribution was also performed on each representative structure, indicating that M6 (-26.32 kcal/mol), M7 (-35.62 kcal/mol), M9 (-29.98 kcal/mol), M10 (-42.73 kcal/mol), M14 (-44.47 kcal/mol), and M15 (-35.66 kcal/mol) were better than others. Moreover, the ligand always needed to deform from the solution before binding with the protein, so the conformational energy penalty of each conformer was also important. Hence, the conformational energies of each conformer were calculated by using Gaussian 03 at 6-31+G* basis set with the B3LYP method plus PCM solvation model. It had been expected that the bioactive conformations for highly active molecules had conformational energy penalties of less than ~6 kcal/mol [4]. The favorable binding mode was selected to be the one which has both the lowest binding energy, the reasonable orientation for reaction, and lower conformational energy penalty. The conformational penalty of M6 is 7.06 kcal/mol, which is a little higher. And the orientations for reaction of M7, M9, and M10 are not proper, because of longer distance between the reaction centers. Synthetically taking these factors into account, M14 and M15 are more reasonable binding mode to be further analysed.

| Comformations | Autodock4 binding energy (kcal/mol) | MM-PBSA binding energy (kcal/mol) | Reaction Center Distance (Å) | Conformational energy penalty (kcal/mol)^a^ |
| --- | --- | --- | --- | --- |
| M1 | -8.25 | 13.26 | 6.79 | 0.00 |
| M2 | -11.03 | -17.22 | 3.17 | 3.09 |
| M3 | -10.76 | -26.01 | 3.06 | 0.18 |
| M4 | -8.47 | -25.82 | 2.96 | 6.27 |
| M5 | -10.23 | 5.16 | 5.07 | 3.24 |
| M6 | -9.68 | -26.32 | 3.18 | 7.06 |
| M7 | -9.85 | -35.62 | 4.72 | 4.36 |
| M8 | -9.43 | -3.87 | 3.88 | 6.92 |
| M9 | -8.86 | -29.98 | 4.50 | 3.91 |
| M10 | -9.53 | -42.73 | 4.70 | 8.48 |
| M11 | -8.35 | -11.00 | 3.84 | 9.60 |
| M12 | -10.45 | 0.22 | 4.49 | 6.97 |
| M13 | -9.47 | 1.48 | 3.06 | 0.93 |
| M14 | -10.99 | -44.47 | 3.05 | 2.82 |
| M15 | -11.88 | -35.66 | 3.07 | 5.78 |
| M16 | -12.48 | -19.80 | 3.39 | 2.71 |
| M17 | -11.20 | -24.52 | 3.93 | 7.58 |
| M18 | -11.18 | -7.57 | 3.63 | 8.49 |

*^a^*Conformer-1 is around the global minimum conformation. The conformational energy penalties of each conformer is defined by the difference with conformer-1.
