# Supplementary material for: Computational and Experimental Insights into the Mechanism of Substrate Recognition and Feedback Inhibition of Protoporphyrinogen Oxidase
Source: PLoS One. 2013 Jul 23;8(7):e69198. doi: 10.1371/journal.pone.0069198 (PMC3720618; doi:10.1371/journal.pone.0069198)
Supplement: Table S2 — Energetic results of MM-PBSA calculation based on MD sampling and the corresponding convergence analysis. (DOC) [file pone.0069198.s011.doc]

***Table S2*:** Energetic results of MM-PBSA calculation based on MD sampling and the corresponding convergence analysis. In order to further evaluate the convergence of the MD simulation and binding free energy calculation, snapshots from different intervals of the last 1 ns trajectory were compared by performing the MM-PBSA calculation. For M14, the lowest binding free energy was -1.11 kcal/mol based on 100 snapshots and the highest binding free energy was 0.18 kcal/mol based on 10 snapshots, the difference of which was 1.29 kcal/mol. For M15, the lowest binding free energy was -15.95 kcal/mol based on 100 snapshots and the highest binding free energy was -13.86 kcal/mol based on 5 snapshots, the differences of which was 2.09 kcal/mol. The energy differences were in a reasonable error range, indicating the equilibration of the MD simulations. We used the calculated binding free energies of -1.01 kcal/mol for M14 and -15.52 kcal/mol for M15 based on large sampling of 1000 snapshots. Further, MM-PBSA calculations based on the repeated MD trajectory of M14 and M15 (M14R and M15R) were consistent with the above. The binding free energy is -0.80 kcal/mol for M14Repeat and -14.58 kcal/mol for M15Repeat. The energy differences were smaller than 1 kcal/mol with the previous, demonstrating that the MD simulation and MM-PBSA calculation were stable and would not changed with different sets of parameters and force-field. According to the components of the binding free energy, the overall electrostatic interaction of M15 (-244.94 kcal/mol) was lower than that of M14 (-215.57 kcal/mol) by ~29 kcal/mol, whereas the overall solvation energy cost of M15 (269.77 kcal/mol) was higher than that of M14 (255.11 kcal/mol) by ~15 kcal/mol, and the entropic cost of M15 (26.62 kcal/mol) was similar with that of M14 (26.39 kcal/mol). The reaction orientation (distances and angle) in the two binding modes were also analyzed. The reaction distance (red dotted line) was defined between the methylene bridge hydrogen atom of protogen and the N_5_ atom of the FAD as following. Compared with M14 (3.48 Å), this distance was a little shorter in M15 (3.30 Å). The reaction angle was defined as following. Only when this angle is close to 180°, the hydrogen atom in *meso* position of protogen is more overlapped with the lone pair electrons of N_5_ atom and hence, the hydrogen transfer reaction becomes easier. From the above points of view, M15 is more reasonable than M14.

The product-proto binding structure was produced based on M15 and the MD simulation of proto was also performed. Based on the MD trajectory of proto, MM-PBSA calculation was performed (Model: Product). The binding free energy of the product-proto (-28.43 kcal/mol) was lower than that of reactant-protogen (-15.52 kcal/mol), which indicated that the binding of proto was stronger than protogen. This result was consistent with the PMF calculation, demonstrating the rationality of the free energy calculation.

|  | Free energy (Kcal/mol) | | | | | | | | | **Distance_(average)_**  **(Å)** | **Angle_(average)_**  **(degree)** |
| --- | --- | --- | --- | --- | --- | --- | --- | --- | --- | --- | --- |
| Model | snapshot^a^ | △E_ELE_ | △E_VDW_ | △E_MM_ | △G_solv_ | △E_bind_ | -T△S | △G_bind_ | **ΔG_bind(average)_** |
| M14 | 5 | -220.13 | -65.07 | -285.21 | 257.58 | -27.63 | 27.23 | -0.40 (4.89)^b^ | **-0.68 (1.71)** | **3.48 (0.36)** | **88.60 (4.24)** |
|  | 10 | -216.36 | -65.87 | -282.23 | 255.70 | -26.53 | 26.71 | 0.18 (3.00) |
|  | 20 | -213.49 | -66.78 | -280.27 | 254.00 | -26.27 | 26.10 | -0.17 (2.28) |
|  | 40 | -215.00 | -66.77 | -281.77 | 254.91 | -26.86 | 26.01 | -0.85 (1.52) |
|  | 50 | -216.51 | -66.41 | -282.92 | 255.24 | -27.68 | 26.62 | -1.06 (1.46) |
|  | 100 | -215.88 | -66.81 | -282.69 | 255.31 | -27.39 | 26.28 | -1.11 (0.97) |
|  | 200 | -216.02 | -66.82 | -282.84 | 255.60 | -27.34 | 26.36 | -0.98 (0.64) |
|  | 500 | -215.55 | -66.93 | -282.49 | 255.38 | -27.11 | 26.39 | -0.72 (0.40) |
|  | 1000 | -215.57 | -66.94 | -282.51 | 255.11 | -27.40 | 26.39 | -1.01 (0.27) |
| M14R^c^ | 100 | -214.60 | -68.64 | -283.24 | 257.16 | -26.08 | 25.28 | -0.80 (0.47) |
| M15 | 5 | -247.32 | -66.66 | -313.98 | 272.70 | -41.28 | 27.42 | -13.86 (1.52) | **-15.31 (0.92)** | **3.30 (0.27)** | **140.08 (5.79)** |
|  | 10 | -249.07 | -67.63 | -316.69 | 274.48 | -42.21 | 26.58 | -15.63 (1.92) |
|  | 20 | -247.12 | -66.73 | -313.84 | 272.48 | -41.36 | 26.66 | -14.70 (1.30) |
|  | 40 | -245.87 | -66.86 | -312.72 | 271.09 | -41.64 | 26.69 | -14.95 (1.07) |
|  | 50 | -245.88 | -66.47 | -312.35 | 270.03 | -42.32 | 26.50 | -15.82 (0.89) |
|  | 100 | -246.49 | -66.54 | -313.03 | 270.60 | -42.43 | 26.48 | -15.95 (0.61) |
|  | 200 | -245.24 | -66.84 | -312.08 | 269.74 | -42.34 | 26.57 | -15.77 (0.45) |
|  | 500 | -245.26 | -66.82 | -312.09 | 269.93 | -42.16 | 26.59 | -15.57 (0.29) |
|  | 1000 | -244.94 | -66.97 | -311.91 | 269.77 | -42.14 | 26.62 | -15.52 (0.21) |
| M15R | 100 | -241.02 | -67.61 | -308.63 | 267.72 | -40.91 | 26.33 | -14.58 (0.38) |
| Product | 100 | -240.01 | -58.76 | -298.77 | 248.09 | -50.69 | 22.26 | -28.43 (0.38) |  |  |  |

*^a^* The number of samples for the MM-PBSA calculation. *^b^* Number in the parenthesis means standard error. *^c^* Means M14_Repeat and M15_Repeat.
